# Tendons and ligaments of the *Rangifer tarandus* metapodial and hoof

**DOI:** 10.1007/s00300-021-02919-z

**Published:** 2021-07-26

**Authors:** Emily Hull, Mitchell Semeniuk, Hanna-Leena Puolakka, Sanna-Mari Kynkäänniemi, Sirpa Niinimäki

**Affiliations:** 1 University of Oulu, Oulu, Finland; 2 University of Alberta, Edmonton, Canada

**Keywords:** Rangifer tarandus, Gross anatomy, Hoof, Phalanges, Metapodials

## Abstract

*Rangifer tarandus*, the northern species including both reindeer and caribou, is a pillar of northern ecosystems and the lives of northern peoples. As the only domestic cervid, reindeer are important not only to the herders and hunters who presently interact with them, but also to zooarchaeologists and palaeontologists tracing their histories. Unfortunately, limited anatomical information on *Rangifer tarandus* muscles is available beyond descriptions of the large muscle groups. The lower limb and hoof in particular is poorly documented. This is problematic, as this important body part has the potential to be informative in zooarchaeological analyses of habitual activity, especially in regards to historical animal health, movement, and habitual activity. Better understanding of the hoof can additionally be useful to herders and veterinarians seeking to provide veterinary care for living animals. This study has used dissections and comparisons of the reindeer hoof with other domestic ungulates to document both the common and unique structures in *Rangifer tarandus* hooves, including the presence and attachment points of these structures. As these structures have proved unique, especially in regards to the dewclaw, it is important that other ungulates not be used exclusively in the analysis of *Rangifer tarandus* remains.

## Introduction

While reindeer and caribou are and have been a keystone species for human survival and ecological stability in the Arctic and sub-Arctic north, gaps in our knowledge of *Rangifer tarandus* anatomy persist. This is particularly true when discussing the hooves of reindeer. Veterinarians, hunters, and herders have first-hand knowledge of the anatomy and physiology of the reindeer hoof, but no guide for the novice has yet been produced. This study presents the origins, insertions, and primary actions of the tendons and ligaments of the hoof in order to further knowledge of *Rangife*r biology and anatomy.

In human osteology, osseous changes at the points of muscle, tendon, or ligament insertion, known as entheseal changes, are commonly examined to help reconstruct habitual activity (Villotte and Knüsel 2013; Wilczak 1998; Molnar 2005). This technique, in conjunction with paleopathology, is also beginning to be used in zooarchaeology in determining the habitual activities of domestic animals, particularly those of reindeer (Hull et al. 2020, 2021, Niinimäki and Salmi 2016; Salmi and Niinimäki 2016; Telldahl 2012, Thomas 2008). In northern archaeology and human-animal studies, reindeer have been the subject of study as prey animals, herded animals, penned animals, ridden mounts, draught animals, and ritual offerings (Aaris-Sørensen et al. 2007; Ingold 1980; Mirov 1945; Nyyssönen and Salmi 2013; Salmi et al. 2015; Sommerseth 2011; Stammler and Takakura 2010, Stépanoff et al. 2017, Willerslev et al. 2015). Given this wide array of potential roles played by reindeer, determining the activities they undertook in life will help scholars better discern the specific ways in which these animals interacted with humans and their broader environments.

The study of entheseal changes as an analytical technique is predicated on the knowledge of the exact muscles, tendons, and ligaments that act to cause habitual stress on the bone. Without this baseline information, accurate and effective assessments of habitual activity are impossible. In order to glean information from the entheseal changes of the phalanges, for example, it is first paramount to understand the soft tissues structures of the hoof and their functions. An exploration of the effects of entheseal change is not possible without an understanding of what tendons and ligaments are acting upon bone to develop these changes, and the actions that produce these changes are not possible to investigate without knowing what muscles, and therefore which actions, produce stress at an entheses.

## Background and reference species

*Rangifer tarandus* is a northern species with many subspecies and uniquely adapted ecotypes. Known as reindeer in Eurasia and caribou in North America, this species has been an important prey animal for humans since the Magdalenian, c. 17000–12000 before present (Kuntz and Costamagno 2011). Reindeer have become domestic herd animals who provide meat and fur, but also locomotion, traction, and milk (Aaris-Sørenson et al. 2007; Andersen 2011; Banfield 1961; Bjørnstad et al. 2012; Laufer 1917; Mirov 1945). Reindeer are of great interest to zooarchaeologists tracing cultural change and incipient domestication, as reindeer have held differing roles within human societies throughout human and animal history, and populations and behaviors have shifted along with these changes (Bjørnstad et al. 2012; Hedman et al. 2015; Helskog 2011; Laufer 1917; Sommerseth 2011; Willerslev et al. 2015). While reindeer are far from the only domestic ungulate, they are the only domestic cervid. Even among cervids, reindeer hoof morphology is unique. While many studies of the reindeer skeleton have been conducted, most studies of *Rangifer tarandus* musculoskeletal anatomy focus on the torso and upper limbs, as these are the most well-known and valuable areas of musculature to hunters and butchers (Hull et al. 2020; Nieminen and Helle 1980; Niinimäki and Salmi 2016, Niinimäki et al. 2019; Pelletier et al. submitted, Puputti and Niskanen 2008; Salmi and Niinimäki and Salmi 2016; Salmi et al. 2020). The most complete guide to the musculoskeletal anatomy of the *Rangifer tarandus* limbs is Wareing et al. (2011), which identifies and defines the gross anatomy of the limbs. However, the discreet attachment point of tendon insertion and ligamentory architecture of the lower limbs are not described in enough detail that points of insertion and connection on the phalanges can be determined, (and are presented below in Table 1.) Additionally, there are no muscles present in the metapodial or hoof, as their movements are affected by muscles closer to the body and attached by these tendons. A precise and detailed guide to the soft tissue of the *Rangifer tarandus* lower limb and hoof is therefore warranted.

**Table 1:** Origins and insertions

Metacarpus	Origin	Insertion	Action
Superficial digital flexor tendon	Medial epicondyle of humerus (Wareing et al. 2011)	Flexor tuberosities of PII	Digital flexion; Forms **manicae flexoriae** around deep dig. flexor
Deep digital flexor tendon	Medial epicondyle of humerus (Wareing et.al. 2011)	Flexor tubercle of PIII	Flexion of the hoof
Interosseous ligaments	Proximal dorsal aspect of metacarpal		
Interosseii III (medial)			
Axial extensor branch	Interosseous III	Extensor junction of the dorsal pastern joint/extensor tuberosity of PII	Extension and adduction of the digits
Abaxial extensor branch	Interosseous III	Extensor junction of the dorsal pastern joint/extensor tuberosity of PII/soleal border of PIII	Extension and abduction of the digits
Interosseous accessory branch to paradigitii II	Interosseous III	Axial aspect of MCII/proximal abaxial surface of PI of digit III	Stabilization and prevention of over-abduction of the dewclaw
Interosseii IV (lateral)			
Axial extensor branch	Interosseous IV	Extensor junction of the dorsal pastern joint/ extensor tuberosity of PII	Extension and adduction of the digits
Abaxial extensor branch	Interosseous IV	Extensor junction of the dorsal pastern joint/extensor tuberosity of PII/soleal border of PIII	Extension and abduction of the digits
Interosseous accessory branch to paradigitii V	Interosseous IV	Axial aspect of MCV/proximal abaxial surface of PI of digit IV	Stabilization and prevention of over-abduction of the dewclaw
Lateral digital extensor (runs jointly w/med. dig. ext., bifurcates at fetlock joint.) Extensor digitii quarti propruis	Lateral epicondyle of humerus (Wareing et.al. 2011)	Extensor junction of the dorsal pastern joint/dorsal border of PIII	Extension of the digits
Common digital extensor	Lateral epicondyle of humerus (Wareing et.al. 2011)	Extensor process of PIII	Extension of the digits
Medial digital extensor (runs jointly w/lat. dig. ext., bifurcates at fetlock joint.) Extensor digitii tertii propruis	Lateral epicondyle of humerus (Wareing et.al. 2011)	Extensor junction of the dorsal pastern joint/extensor tuberosity of PII/axial border of PIII	Extension of the digits
Plantar annular ligament	Surrounds fetlock joint		Supports fetlock joint
Proximal sesamoidean collateral ligaments			
Collateral ligaments of the proximal sesamoids	Intercapital notch of MC; concavities of metacarpal concavities	Abaxial aspects of proximal sesamoids	Connection and support of proximal sesamoids to the fetlock joint
Cruciate ligaments of the proximal sesamoids	Axial aspects of proximal sesamoids	Axial aspects of opposite proximal sesamoids	Connection and support of proximal sesamoids to the fetlock joint
Proximal annular ligament	Proximal palmar eminences of PI	Proximal palmar eminences of PI	Connection of flexor tendons to PI
Distal annular ligament	Distal palmar eminences of PI	Distal palmar eminences of PI	Connection of flexor tendons to PI
Proximal accessory ligament of paradigitii II and V	Axial portion of paradigital PI	Extensor junction of the dorsal pastern joint/extensor tuberosity of PII	Extension of the dewclaw
Distal accessory ligament of paradigitii II and V	Axial portion of paradigital PI	Parietal groove of PIII	Flexion of the dewclaw
Metacarpophalangeal collateral ligaments (axial and abaxial)	Axial and abaxial aspects of trochanters of metacarpal capita	Lateral aspects of proximal PI	Support and connection of fetlock joint
Metacarpophalangeal collateral ligaments (medial)	Intercapital notch of metacarpal capita	Medial aspects of proximal PI	Support and connection of fetlock joint
Proximal interphalangeal collateral ligaments (axial and abaxial)	Axial and abaxial concavities on the head of PI	Concavity in the flexor tuberosity of PII	Support and connection of pastern joint
Distal interphalangeal collateral ligaments (axial and abaxial)	Axial and abaxial concavities on the head of PII	Dorsal surface of PIII	Support and connection of coffin joint
Distal interphalangeal cruciate ligaments	Proximal and distal medial surfaces of PII	Proximal and distal medial surfaces of PII	Support and connection of coffin joint
Distal collateral sesamoidean ligament	Deep digital flexor tendon	Plantar surface of proximal distal sesamoid	Connection of distal sesamoid/navicular bone and support of coffin joint
Impar ligament	Plantar surface of distal sesamoid below PIII articular surface	Plantar surface of PIII below sesamoidal articular surface	Connection of distal sesamoid/navicular bone and support of coffin joint

Superficial digital flexor tendon	Supracondylar fossa of femur	Flexor tuberosity of PII	Digital flexion; Forms **manicae flexoriae** around deep dig. flexor
Deep digital flexor tendon	Lateral condyle of tibia	Flexor tubercle of PIII	Digital flexion
*Interosseous ligaments*	Proximal dorsal aspect of metatarsal		
Interosseii III			
Axial extensor branch	Interosseii III	Extensor junction of the dorsal pastern joint/extensor tuberosity of PII	Digital extension and adduction
Abaxial extensor branch	Interosseii III	Extensor junction of the dorsal pastern joint/extensor tuberosity of PII/ soleal border of PIII	Digital extension and abduction
Interosseous accessory branch to paradigitii II	Interosseii III	Axial aspect of PI of paradigitii II	Stabilization and prevention of over-abduction of the dewclaw
Interosseii IV			
Axial extensor branch	Interosseii VI	Extensor junction of the dorsal pastern joint/extensor tuberosity of PII	Digital extension and abduction
Abaxial extensor branch	Interosseii VI	Extensor junction of the dorsal pastern joint/extensor tuberosity of PII/soleal border of PIII	Digital extension and abduction
Interosseous accessory branch to paradigitii V	Interosseii VI	Axial aspect of PI of paradigitii V	Stabilization and prevention of over-abduction of the dewclaw
Lateral digital extensor (runs jointly w/ med. dig. ext., bifurcates at fetlock joint)	Caudomedial aspect of proximal tibia (Wareing et al. 2011)	Extensor junction of the dorsal pastern joint/extensor tuberosity of PII/ dorsal border of PIII	Digital extension of digit III
Long digital extensor	Lateral condyle of tibia *m. peroneus longus* (Wareing et al. 2011)	Extensor process of PIII	Digital extension
Medial digital extensor (runs jointly w/ med. dig. ext., bifurcates at fetlock joint.)	Lateral condyle of tibia *m. peroneus longus* (Wareing et al. 2011)	Extensor junction of the dorsal pastern joint/extensor tuberosity of PII/axial border of PIII	Digital extension of digit IV
Plantar annular ligament	Surrounds fetlock joint	Surrounds fetlock joint	
Proximal sesamoidean collateral ligaments			
Collateral ligaments of the proximal sesamoids	Intercapital notch of MC; concavities of metacarpal concavities	Abaxial aspects of proximal sesamoids	Connection and support of proximal sesamoids to the fetlock joint
Cruciate ligaments of the proximal sesamoids	Axial aspects of proximal sesamoids	Axial aspects of opposite proximal sesamoids	Connection and support of proximal sesamoids to the fetlock joint
Proximal annular ligament	Proximal palmar eminences of PI	Proximal palmar eminences of PI	Connection of flexor tendons to PI
Distal annular ligament	Distal palmar eminences of PI	Distal palmar eminences of PI	Connection of flexor tendons to PI
Proximal accessory ligament of paradigitii II and V	Axial portion of paradigital PI	Extensor junction of the dorsal pastern joint	Extension of the dewclaw
Distal accessory ligament of paradigitii II and V	Axial portion of paradigital PI	Parietal groove of PIII	Flexion of the dewclaw
Metatarsophalangeal collateral ligaments (axial and abaxial)	Axial and abaxial aspects of trochanters of metatarsal capita	Lateral aspects of proximal PI	Support and connection of fetlock joint
Metatarsophalangeal collateral ligaments (medial)	Intercapital notch of metatarsal capita	Medial aspects of proximal PI	Support and connection of fetlock joint
Proximal interphalangeal collateral ligaments (axial and abaxial)	Axial and abaxial concavities on the head of PI	Concavity in the flexor tuberosity of PII	Support and connection of pastern joint
Distal interphalangeal collateral ligaments (axial and abaxial)	Axial and abaxial concavities on the head of PII	Dorsal surface of PIII	Support and connection of coffin joint
Distal interphalangeal cruciate ligaments	Proximal and distal medial surfaces of PII	Proximal and distal medial surfaces of PII	Support and connection of coffin joint
Distal collateral sesamoidean ligament	Deep digital flexor tendon	Plantar surface of proximal distal sesamoid	Connection of distal sesamoid/navicular bone and support of coffin joint
Impar ligament	Plantar surface of distal sesamoid below PIII articular surface	Plantar surface of PIII below sesamoidal articular surface	Connection of distal sesamoid/navicular bone and support of coffin joint

Though a small portion of the overall body mass, *Rangifer tarandus* hooves are of primary importance to their health and viability. The foot is the interface between the environmental substrate and the body, but for the reindeer, the hoof functions in many other important ways. As a migratory species, *Rangifer tarandus* must depend upon their hooves to carry them long distances (Ferguson and Elkie 2004). Further, their cold-weather adaptation requires strong forelimb digging, which involves using the front hooves to shovel through dense snowpack for lichens during snowy seasons (Formozov 1946; Nieminen 1994; Takatsuki 1992; Telfer and Kelsall 1979, 1984).

The far northern environments that constitute the habitat of most *Rangifer tarandus* have produced evolutionary adaptations to extreme cold weather and winter foraging. These include foraging adaptations to both tundra and the deep snow drifts of the taiga, both of which necessitate a broad hoof platform. Most studies of *Rangifer tarandus* locomotion measure hoof-load (Telfer and Kelsall 1979, 1984; Takatsuki 1992), which provides a proportional measurement of body weight to foot area. The smaller the hoof-load, the higher the proportion of foot area to overall body mass. Caribou and reindeer have a much lower hoof-load than other ungulates, including *Odocoileus virginianus* and *Odocoileus hermonius*, a size ratio more akin to that of northern predator species than prey (Telfer and Kelsall 1979, 1984; Blanco and Gambini 2006). Their broad feet allow reindeer and caribou to walk over the snow, and their strong digging hooves can penetrate snowpack and deep, dense snow drifts. More southerly ecotypes such as boreal reindeer and caribou often wade through muddy waters and swamplands, and navigate rocky and rugged terrain, again depending on their broad hoof-spread to facilitate mobility and foraging in these environments (Formozov 1946, Nieminen 1990, 1994; Takatsuki 1992, Telfer and Kelsall 1971, 1979, 1984).

## Bones of the lower limb

**Fig. 1:**
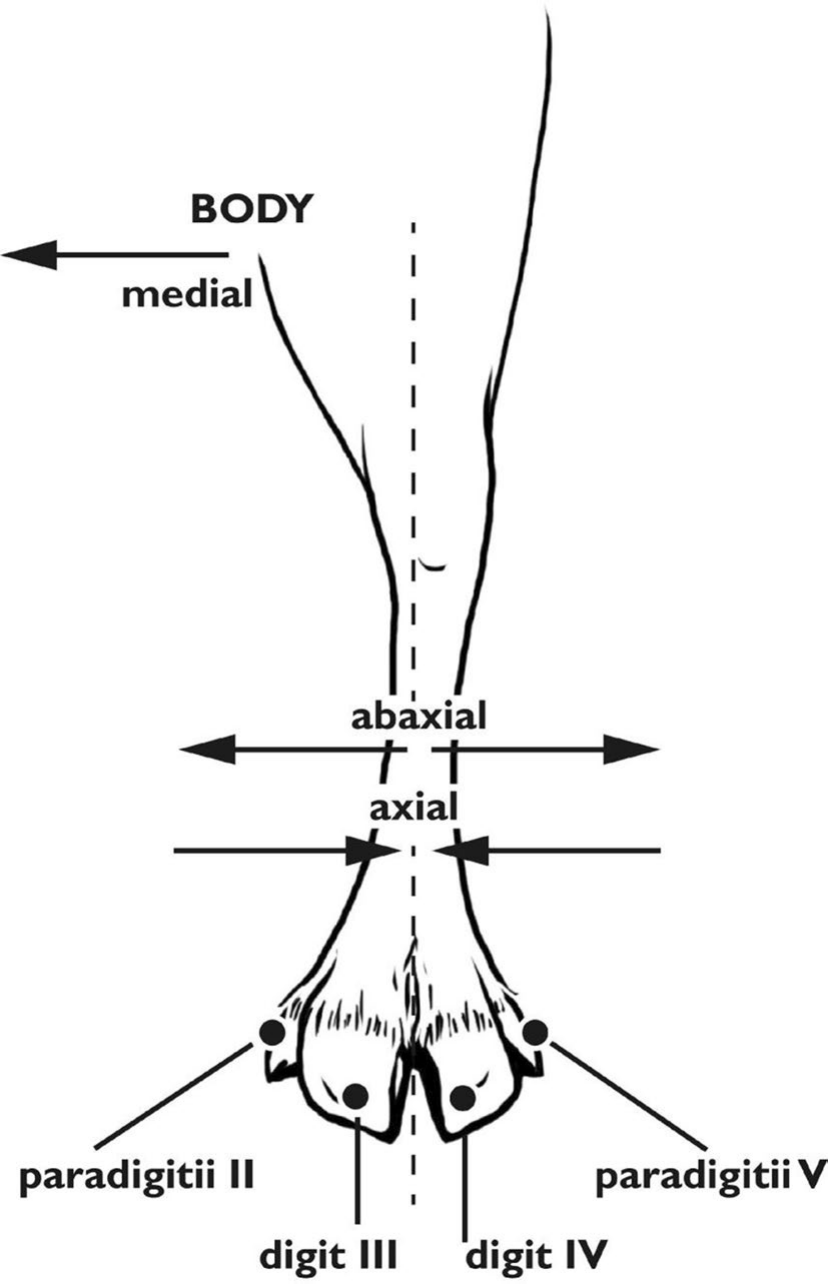
Directions and terminology. Left forelimb pictured. Illustration by E. Hull

**Fig. 2:**
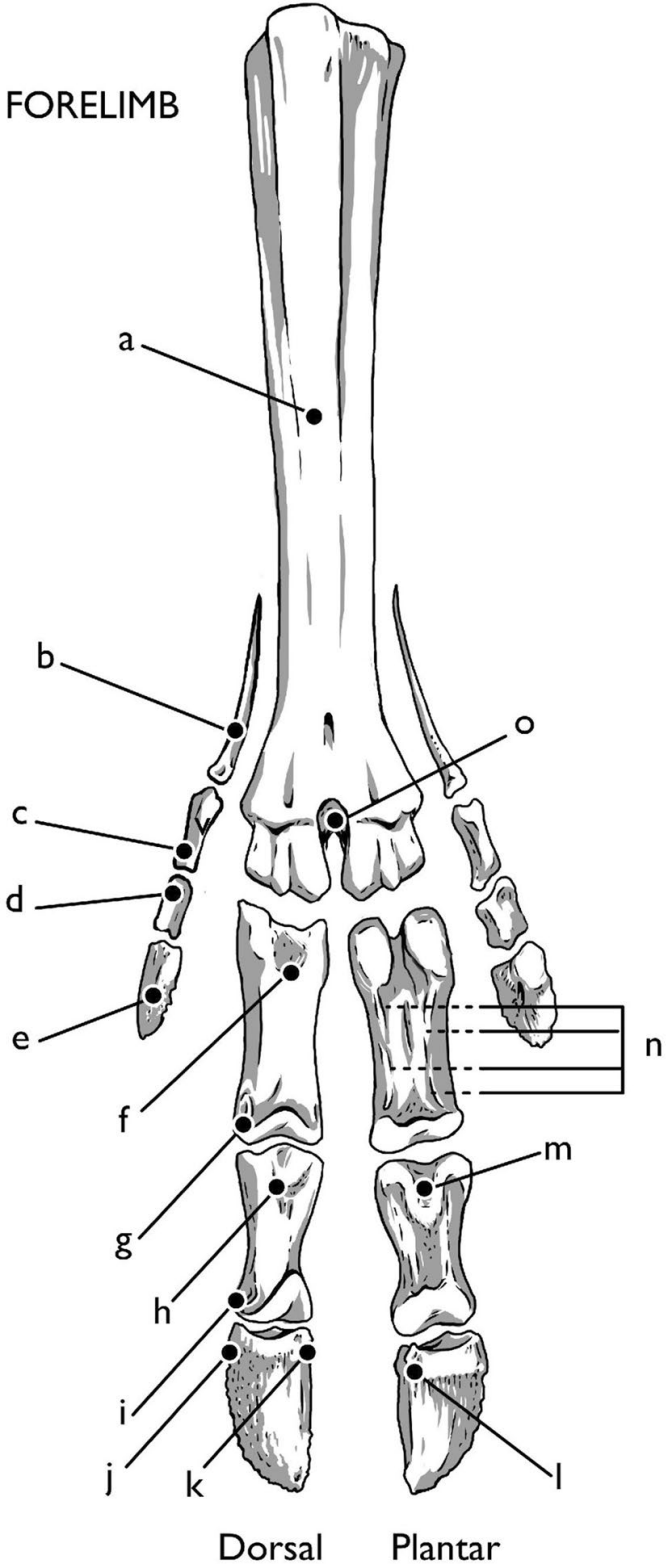
Bones of the forelimb. **a** metacarpal (only dorsal aspect shown), **b** MC V/II, **c** paradigitii PI, **d** paradigitii PII, **e** paradigitii PIII, **f** first phalanx (PI), **g** head of PI, **h** second phalanx (PII), **i** head of PII, **j** third phalanx (PIII), **k** extensor process, **l** flexor tubercle, **m** flexor tuberosity, **n** axial and abaxial palmar eminences, **o** intercapital notch. Illustration by E. Hull

**Fig. 3:**
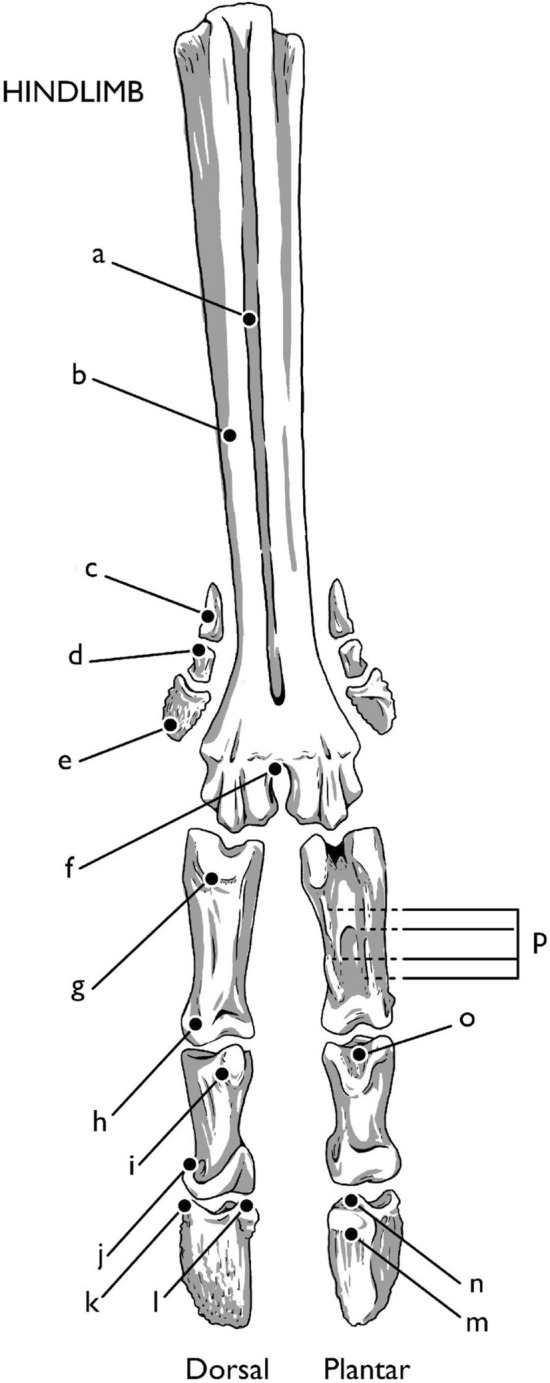
Bones of the hindlimb. **a** longitudinal groove, **b** metatarsal (only dorsal aspect shown), **c** paradigitti PI, **d** paradigitti PII, **e** paradigitti PIII, **f** intercapital notch, **g** first phalanx (PI), **h** head of PI, **i** extensor tubercle of second phalanx (PII), **j** head of PII, **k** third phalanx (PIII), **l** extensor process, **m** plantar surface, **n** flexor tubercle, **o** concavity of flexor tuberosity, **p** axial and abaxial palmar eminences. Illustration by E. Hull

*Rangifer tarandus* are artiodactyls, having two toes and two dewclaws per hoof, with a fused metacarpal/metatarsal III and IV to form the metapodial. Vestigial metacarpal II and V are present as stylets of the forelimb dewclaws, but absent in the hindlimb. Each limb contains (Figs. 1, 2, 3) a metapodial, three phalanges (three per each of two digits: PI, PII, PIII), dewclaws (each containing 4 bones in the forelimb and 3 in the hind), four proximal sesamoids, two distal sesamoids, and, in the forelimb, two dorsomedial sesamoids (Hull 2019). This osteology follows the pattern of other members of Telemetacarpalia (Nieminen 1994). Bovids, and artiodactyls in general, follow the same basic pattern, with the deviation of the dewclaws. In *Bos taurus*, the dewclaws rarely contact the ground, providing a different function than those of cervids. The dewclaws of *Rangifer tarandus* spread out against the ground, providing additional surface area for walking. When the forelimb is flexed, the dewclaws curl inward, providing a more efficient, shovel-shaped plantar surface for digging in the snow.

This increased functionality of the dewclaws leads to more substantial bones of the dewclaw, as well as more extensive ligamentary structures. Because of this, the suspensory ligaments connecting the dewclaw of *Rangifer tarandus* to the hoof proper should be considered as important as the other ligaments. While veterinary literature often glosses over the dewclaws of *Bos taurus*, they must be thoroughly discussed in *Rangifer tarandus* hoof anatomy.

## Materials and methods

Dissections were conducted on the forelimbs and hindlimbs of six individuals: an adult male, an adult female, and four juvenile yearling male *Rangifer tarandus tarandus*. Habitat of the adult reindeer was situated 250 m above sea level in the boreal forest area of central Finland. In summer, reindeer in this herd prefer wetlands, swamp areas between valleys, lake shores, and river shores. In autumn and winter, these reindeer graze in the forest seeking for mushrooms, arboreal lichens, and digging terrestrial lichens and plants under the snow. The adult reindeer also gained supplementary feed in winter. This life history must be considered in examining the robusticity of soft tissue structures.

Dissection began at the proximal metapodial and continued throughout the hoof. Each specimen was first skinned, and dissection proceeded with each compartment, moving from superficial to deep. All dissections were photographed and video-recorded for reference. As the musculature of the fore- and hindlimb have been detailed by Wareing et al. (2011), the dissections and study began distal to the carpals and tarsals, including the metapodials, phalanges, and dewclaws. Results are broken down by joint, with discussions focusing on the component structures of the fetlock, pastern, and coffin joints. As these structures are generally analogous between fore- and hindlimb, the use of domestic ungulate joint terminology is used, except in those places where the metacarpophalangeal joints differ from the metatarsophalangeal joints. Despite the importance of these hoof adaptations to *Rangifer*, no comprehensive guide to the soft tissue structures of the reindeer hoof is available. Other cervids suffer from the same gap in published information. This study, therefore, takes references from better documented species, particularly *Bos taurus*, domestic cattle. Bovids are the most thoroughly documented domestic two-toed ungulates, and as such, bovid literature will provide the primary source for structure identification, nomenclature, and comparative physiology. Specifically, soft tissue terminology was used or adapted from Budras and Habel (2011), Budras et al. (2003), Smallwood (1992), and McLeod (1956), unless otherwise cited. Osteological nomenclature was adopted from Barone (1986), Budras and Habel (2011) and von den Dreisch (1976). Origins and insertions for each tendon and ligament are listed in Table 1.

The ethical implications of animal research are important to the research team, and thus all materials used in this study were considered waste from routine culling. All individuals dissected were culled and donated by reindeer herders. The limbs were unwanted by-products after routine butchering, which would have been thrown out, as there was no useable meat on this portion of the animal. Remains were stored frozen at − 26 °C before the dissection process, then refrigerated throughout. All remains were treated with care, and guidelines for respect toward human cadavers were followed. No animals were killed to facilitate this study, and no animals were purchased for the purposes of study. While this may limit the number of specimens, it follows the ethics set forwards by the research team regarding animal lives.

## The metapodials

The metacarpal (MC) and metatarsal (MT) bones are similar in shape, with a few notable differences (Figs. 2, 3). Both are the fused third and fourth metapodials, and this double-sided form can be seen in the metacarpal from the proximal articular junction with the carpals. Here the tuberosity of MC III designates both the medial side of the metacarpal and division between MC III and MC IV. On the metacarpal, a long dorsal longitudinal groove follows the central line of the shaft, terminating at the intertrochanteric or intercapital notch between the two round articular surfaces of the head of the metacarpal. On the metatarsal, there is no tuberosity of MT III, but both dorsal and ventral longitudinal grooves vertically bisect the metatarsal shaft. The lateral aspect of the dorsal metatarsal shaft, divided by the dorsal longitudinal groove, is significantly more robust than the medial aspect. The interosseous ligaments and flexor tendons run through the dorsal groove the length of the metatarsal (and against the dorsal aspect of the length of the metacarpal), and the combined extensor capsule follows the length of the ventral longitudinal groove on both metapodials.

The round, spool-shaped trochanters on the head of the metapodials provide the platform for articulation of the first phalanx (PI) and the proximal sesamoid. The cup-shaped concavities on the sides of the metapodial heads provide the origin for the abaxial metacarpo- and metatarso-phalangeal collateral ligaments. The deep indentation between the trochanters of the metapodial head are alternately referred to as the intercapital or intertrochanteric groove, which serves as the origin site of the axial metacarpo-/metatarsophalangeal collateral ligaments.

## Metacarpophalangeal joint (forelimb fetlock joint)

The fetlock joint is located where the metapodial articulates with the first phalanx, as well as with the proximal and dorsomedial sesamoids. The metacarpophalangeal joint is held together with collateral ligaments on the abaxial surfaces, axial collateral ligaments between the metacarpal and the first phalanx, and smaller cruciate ligaments supporting the plantar (proximal) sesamoids of each digit. The collateral ligaments of the fetlock joint run from the cup-shaped concavities on the abaxial aspects of the metacarpal capita and then divide, with one ligament going to the proximal plantar surfaces of the first phalanges. The other ligament combines with the cruciate ligaments holding the proximal sesamoids to the first phalanx. The large axial collateral ligaments, which attach the metacarpal to the first phalanges, run from each side of the interior intercapital notch of the metacarpal capita to combine with the axial portion of the small cruciate ligament of the same digit. These small cruciate ligaments, along with small sesamoidean ligaments, attach the metacarpal and proximal sesamoids to the axial eminences along the proximal axial and palmar surfaces of the first phalanx. The fibers of these cruciate ligaments run perpendicularly to the shaft of the first phalanx and may be distinguished by the direction of osseous build-up at the attachment point. This site is adjacent to the attachment point for the proximal annular ligaments, whose attachment site runs along the plantar surface, rather than extending along the axial surface.

**Fig. 4:**
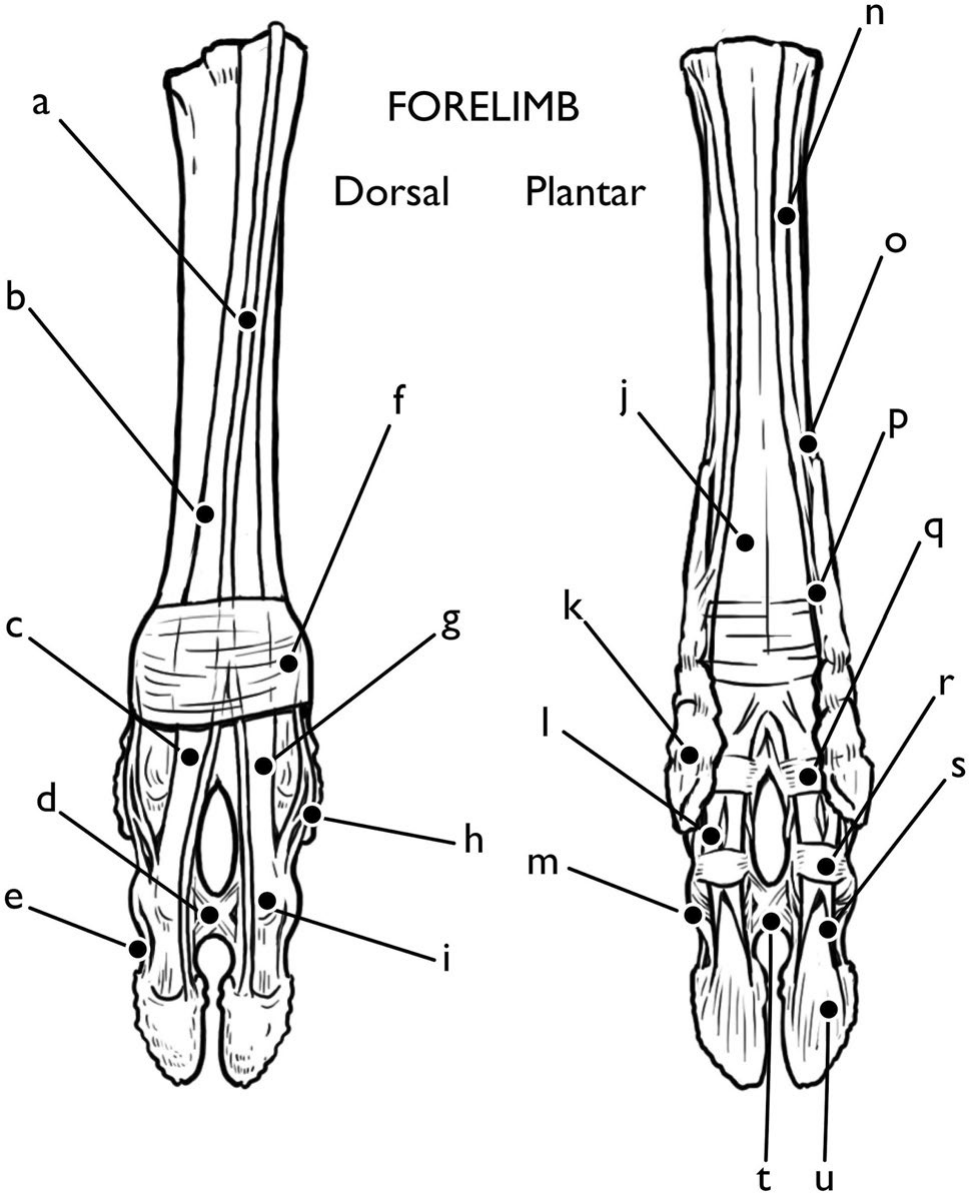
Forelimb tendons and ligaments Dorsal: **a** common digital extensor tendon, **b** medial/lateral extensor tendon, **c** lateral extensor tendon, **d** interdigital cruciate ligament, **e** lateral collateral distal interdigital ligament, **f** plantar annular ligament, **g** medial extensor tendon, **h** abaxial extensor branch, **i** extensor junction plantar, **j** superficial and deep flexor capsule, **k** paradigitii/dewclaw, **l** separation of *manicae flexoriae*, **m** lateral collateral interdigital ligament, **n** interosseous ligament, **o** interosseous accessory branch to paradigitii II/V, **p** proximal accessory ligament to paradigitii II/V, **q** proximal annular ligament, **r** distal annular ligament, **s** superficial flexor tendon insertion, **t** interdigital cruciate ligament, **u** deep flexor tendon insertion. Illustration by E. Hull

Two layers of tendons run down the dorsal side of the metacarpal (Fig. 4). Superficially, the robust and large combined capsule of the superficial and deep flexor tendons run along the metacarpal, with the superficial flexor tendon creating a sheath (*manicae flexoriae*) around the deep flexor tendon until just proximal to the pastern joint. The combined tendons bifurcate at the fetlock joint to follow the individual digits III and IV. The interosseous ligaments run deep to the flexors. The interosseous ligaments begin as a thin, flat, fibrous structure that lays flat against the metacarpal. At the fetlock joint of the forelimb, the interosseous ligament divides into seven branches. The axial and abaxial extensor branches (divided into interosseous III and IV dependant on the digit which they serve) run on either side of each digit, while the medial branch inserts into the intercapital notch of the metapodial. The axial branches run on the internal surface of the hoof, crossing from the plantar to dorsal aspect of the hoof. The abaxial branches follow an analogous path on the outer surface of the hoof. The abaxial branch also subdivides, providing a small tendon to the proximal sesamoid bone. The axial and abaxial extensor branches of the interosseous ligaments recombine on the dorsal surface of the hoof, joining with the medial and lateral extensors at the extensor junction of the pastern joint. Two small accessory ligaments also subdivide, with the interosseous accessory ligament for paradigitii V subdividing from interosseous IV and the accessory ligament for paradigitii II subdividing from interosseous III. These ligaments attach to the axial aspect of the stylets of the dewclaws in the forelimb before continuing to a second attachment site on the proximal abaxial surface of PI proper and act to stabilize the dewclaw.

The proximal sesamoids of the hoof sit against the base of the first phalanx and provide a larger articular surface against which the rounded head of the metacarpal hinges. The entire fetlock joint is encased in a thick band of fascia covering the network of suspensory ligaments, as well as the tendons which pass along the dorsal and plantar sides of this joint. Superficially, the palmar annular ligament of digital flexors holds the tendon of the superficial and deep digital flexors in place, while the thin abaxial extensor branches run deep to this, further supporting the proximal sesamoids. They are held in place by the digital flexors until diverging just distally to the fetlock joint, where they curl around the axial and abaxial surfaces of the first phalanx before joining the other extensor tendons on the dorsal aspect of the digit.

The dorsal compartment of this joint contains far less soft tissue than the plantar, as the belly of the combined superficial and digital flexor tendons are by far the most robust soft structure of the hoof. Along the dorsal portion of the joint there run two layers of extensor tendons, all contained within a single tendon sheath. The common digital extensor tendon is a thin, flat band which runs superficially along the dorsal surface of the metapodial, bifurcating at the fetlock joint into the digital extensor tendons for digits III and IV. Deep to this, the lateral and medial digital extensors run, crossing the joint and housing, in the forelimb, the dorsomedial sesamoid bones, one on each digit. These small bones act as miniature patellae, facilitating the extension of the first phalanx at the metacarpophalangeal joint (Hull 2019). It is speculated that the movement of this bone across the joint which creates the distinctive “clicking” noise that heralds reindeer movement, although further research into live animal physiology is needed.

**Fig. 5:**
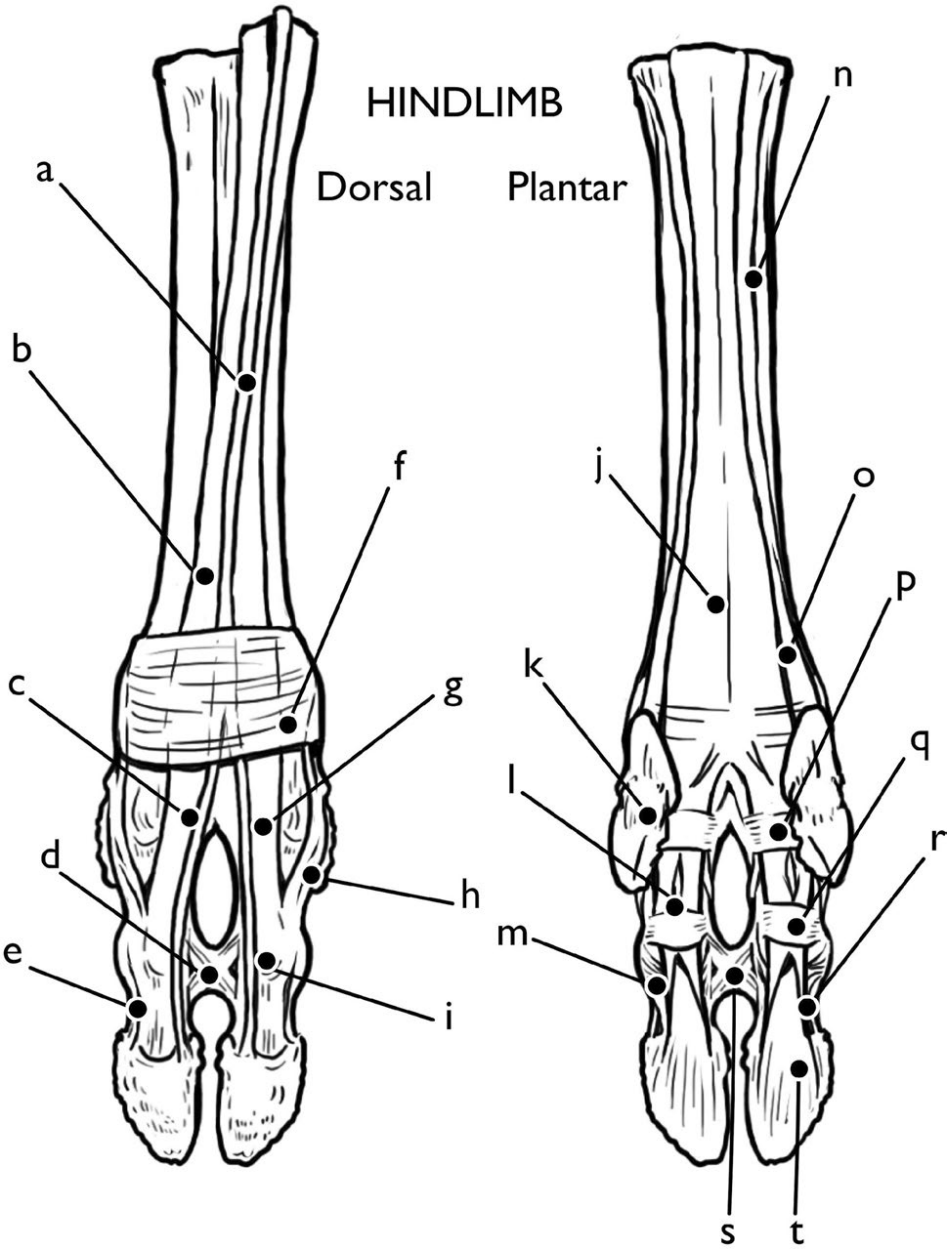
Forelimb, abaxial aspect: **a** lateral digital extensor tendon, **b** sesamoidean ligaments, **c** metacarpophalangeal collateral ligament, **d**, **e**, **f** proximal accessory ligament of paradigitii II/V, **g** extensor junction, **h** abaxial proximal interphalangeal collateral ligament, **i** distal abaxial extensor branch, **j** distal lateral digital extensor tendon, **k** common digital extensor tendon, **l** distal lateral digital extensor tendon insertion, **m** distal accessory ligament of paradigitii II/V, **n** abaxial distal interphalangeal collateral ligament, **o** impar ligament, **p** collateral sesamoidean ligament, **q** deep digital flexor tendon, **r** deep digital flexor tendon, **s** superficial digital flexor tendon/separation of *manicae flexoriae*, **t** distal annular ligament, **u** paradigital distal interphalangeal collateral ligament, **v** proximal annular ligament, **w** combined superficial/deep digital flexor tendon, **x** interosseous ligament. Illustration by E. Hull

**Fig. 6:**
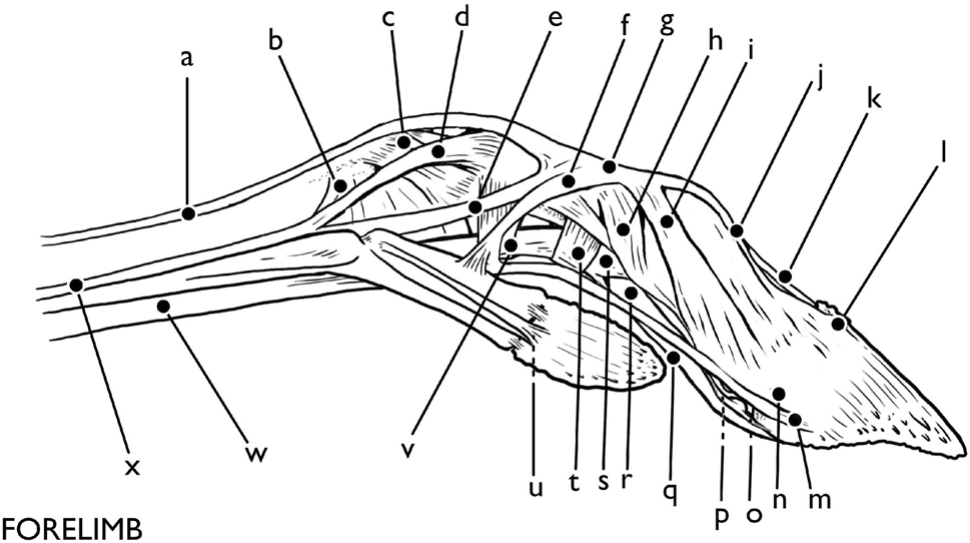
Hindlimb, abaxial aspect: **a** lateral digital extensor tendon, **b** sesamoidean ligaments, **c** metacarpophalangeal collateral ligament, **d** metacarpophalangeal collateral ligament, **e** proximal annular ligament, **f** proximal accessory ligament of paradigitii II/V, **g** extensor junction, **h** abaxial proximal interphalangeal collateral ligament, **i** distal abaxial digital extensor tendon, **j** distal lateral digital extensor tendon, **k** long digital extensor tendon, **l** distal lateral digital extensor tendon insertion, **m** distal accessory ligament of paradigitii II/V, **n** abaxial distal interphalangeal collateral ligament, **o** impar ligament, **p** collateral sesamoidean ligament, **q** deep digital flexor tendon, **r** deep digital flexor tendon, **s** superficial digital flexor tendon/separation of *manicae flexoriae*, **t** distal annular ligament, **u** paradigital distal interphalangeal collateral ligament, **v** combined superficial/deep digital flexor tendon, **w** interosseous ligament. Illustration by E. Hull

**Fig. 7:**
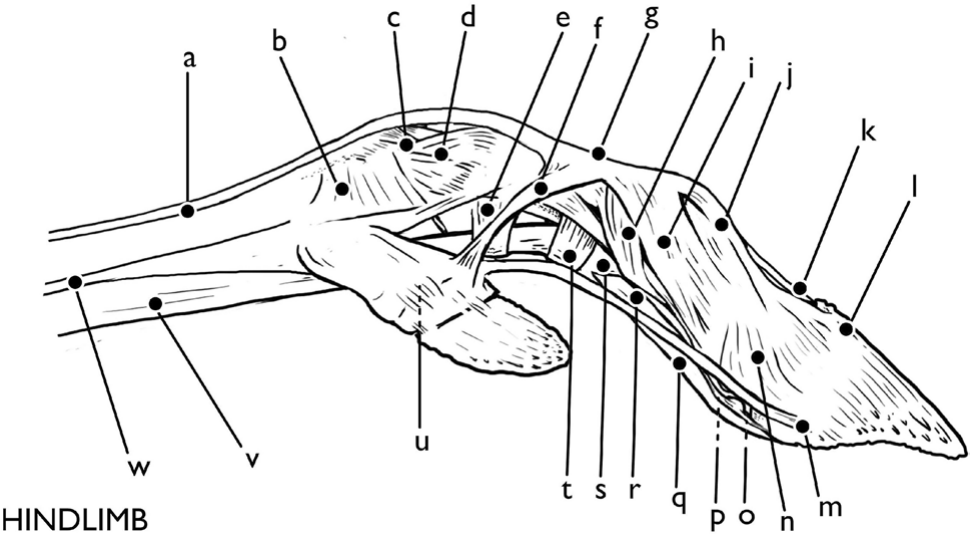
Hindlimb, dorsal view. Left side, hoof capsules removed, right side, hoof capsules present: **a** combined medial/lateral digital extensor ligament, **b** long digital extensor tendon, **c** axial extensor branch, **d** extensor junction, **e** proximal accessory ligament of paradigitii II/V, **f** deep lateral digital extensor tendon, **g**, **h** interphalangeal cruciate ligament, **i** deep lateral digital extensor tendon, **j** distal accessory ligament of paradigitii II/V, **k** distal interphalangeal collateral ligament, **l** proximal accessory ligament of paradigitii II/V, **m**, **n** interosseous accessory branch to paradigitii II/V, **o** axial extensor branch. Illustration by E. Hull

It is also at the fetlock joint that the structure of *Rangifer tarandus* hooves wildly deviate from those of *Bos taurus*. While the tendons and ligaments mentioned above occur in different configurations and slightly different sites, they are nonetheless identifiable as analogous structures. Generally speaking, there are both more in number and more complex suspensory ligaments in the fetlock joint of *Rangifer tarandus* than in *Bos taurus*. These provide flexibility, especially in the abduction of the digits, at the cost of greater stability provided by the thick plantar annular ligament in *Bos taurus*. This is expected considering differing habitats and behaviors. The *Rangifer tarandus* dewclaw, however, has structures without analog in *Bos taurus*, which are described below (Figs. 5, 6, 7).

Proximally, the dewclaws are held in place by both fascia as well as a paradigital accessory branch of interosseous III/IV in the forelimb. This tendon runs the length of the stylet and inserts on the first phalanx of the forelimb dewclaw. Two accessory abaxial ligament branches (the proximal and distal accessory ligaments of paradigitii II/V) also run from the proximal and distal ends of the dewclaw first phalanx. The proximal accessory extensor ligament combines with the abaxial extensor branches, and with the medial and lateral digital extensors, insert at the proximal dorsal aspect of the second phalanx of the digits proper. The action of these ligaments causes the abduction of the dewclaws to extend the digits of the hoof, causing the hoof to splay out, increasing the surface area. The additional distal accessory ligament runs between the distal axial surface of the dewclaw to the proximal abaxial surface of the third phalanx proper, allowing the dewclaws to flex in concert with the other digits of the hoof. They are unable to flex independently. On the forelimb dewclaw, additional small interdigital collateral ligaments run between the small first and second phalanx, mirroring those of the proximal collateral interdigital ligaments on the hoof proper.

Several structures attach to the first phalanx between the fetlock and pastern joints. On the proximal dorsal aspect of the first phalanx, one insertion site of the deep medial and lateral extensor tendons occurs, keeping the dorsomedial sesamoid in place. On the plantar aspect, two important attachment sites produce two bilateral ridges along the shaft of the bone. These ridges are the attachment points for the proximal and dorsal digital annular ligaments, which encircle the strong flexor tendons of the digits. These ligaments are the primary stress points on the first phalanx when the digits are flexed.

## Metatarsophalangeal joint (hindlimb fetlock joint)

The metatarsophalangeal joint is similar to the metacarpophalangeal joint in structure. The collateral and sesamoidal ligaments of the metatarsal follow the same pattern as those of the metacarpal, as do the superficial and deep flexors. The interosseous accessory paradigital ligaments, however, are much more gracile and insert on PI of the dewclaw, as no stylets are present in the hind limb. In the dorsal compartment, the extensors follow a similar pattern, with the long digital extensor running superficially to the medial and lateral extensors, following a path analogous to the common digital extensor of the forelimb.

The tendons of the forelimb dewclaws are more developed than those of the hindlimb, and the forelimb dewclaws are both more robust and contain an additional bone: the vestigial MC II/V, or stylets. Despite this, they retain the same general tendinous and ligamentary structures, with a few important exceptions. Proximally, the dewclaws are held in place by fascia and the digital accessory branch of interosseous III/IV in the forelimb. This ligament runs the length of the stylet and inserts on the first phalanx of the forelimb dewclaw and continues to insert on PI of the digit proper. In the hindlimb, this tendon attaches to and terminates at the first phalanx of the dewclaw. Because of the lack of stylet and reduced size of the first phalanx of the hindlimb dewclaw, the dewclaws are not able to abduct as broadly as in the forelimb. In the forelimb, the proximal accessory ligament of paradigitii II/V runs from the axial surface of the stylet of MC II/V, while in the hindlimb it originates from the proximal aspect of the first phalanx of MT II/V to attach to the extensor junction on PII of the digits proper. The distal accessory ligament of paradigitii II/V remains analogous. While small interdigital collateral ligaments are present on the hindlimb dewclaw, they are less developed than those of the forelimb, and the dewclaw itself has less flexibility between the joints of the dewclaw phalanges.

## Proximal interphalangeal joint of the forelimb and hindlimb (pastern joint)

**Fig. 8:**
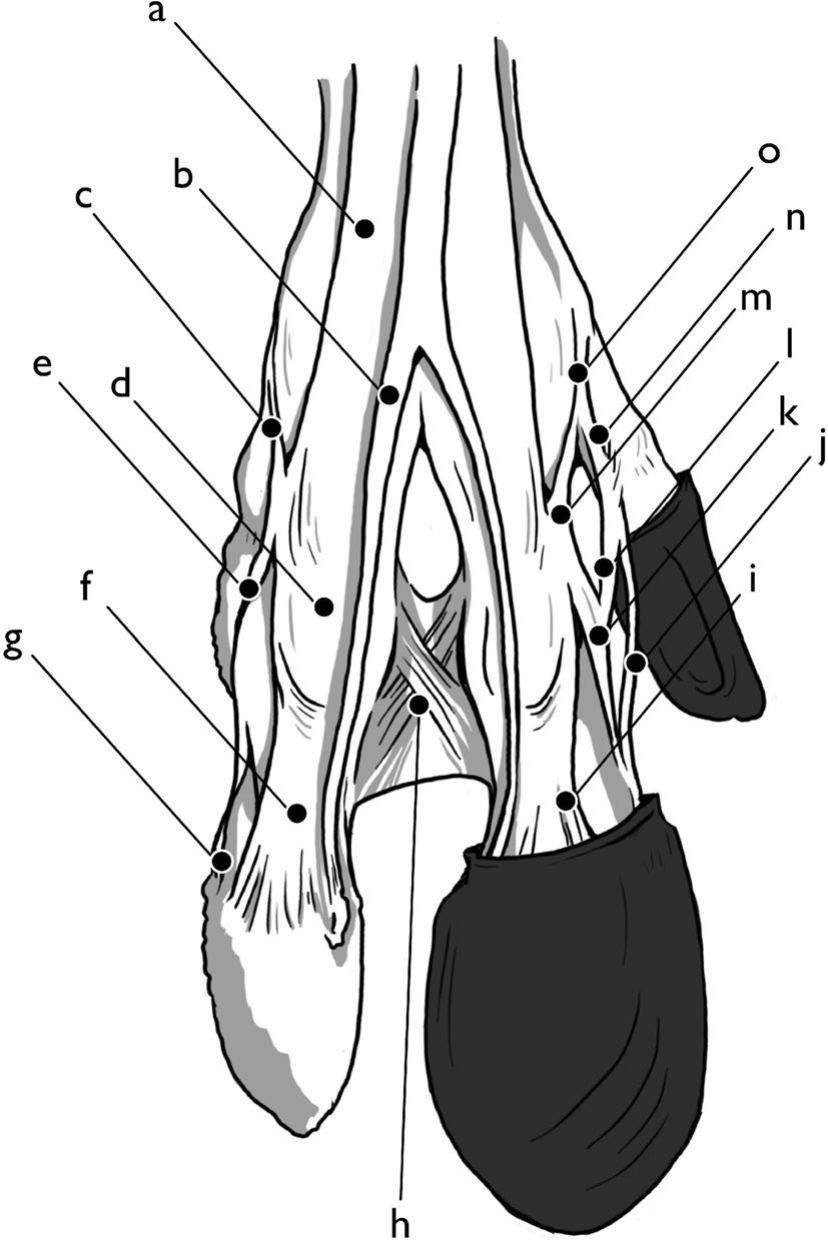
Hindlimb tendons and ligaments Dorsal: **a** long digital extensor tendon, **b** medial/lateral digital extensor tendon, **c** lateral digital extensor tendon, **d** interdigital cruciate ligament, **e** lateral collateral distal interdigital ligament, **f** plantar annular ligament, **g** medial digital extensor tendon, **h** abaxial extensor branch, **i** extensor junction plantar, **j** superficial and deep digital flexor capsule, **k** paradigitii/dewclaw, **l** separation of *manicae flexoriae*, **m** distal interphalangeal collateral ligament, **n** interosseous ligament, **o** interosseous accessory branch to paradigitii II/V, **p** proximal annular ligament, **q** distal annular ligament, **r** superficial digital flexor tendon insertion, **s** interdigital cruciate ligament, **t** deep digital flexor tendon insertion. Illustration by E. Hull

The proximal interphalangeal or pastern joint between the first and second phalanges is less complex than that of the fetlock joint (Figs. 8, 5). Two proximal interphalangeal collateral ligaments run from the axial and abaxial depressions in the head of the first phalanx to insert along the palmar aspect of the second phalanx. Proximal to the pastern joint, the superficial and deep digital flexors run together, with the superficial digital flexor creating a tendinous sheath around the deep digital flexor. Just proximal to the pastern joint, the superficial digital flexor peels away from the deep digital flexor to insert into the flexor tuberosity on the proximal palmar surface of the second phalanx, as the deep digital flexor continues to run along the palmar/plantar aspects of the phalanges.

The medial and lateral digital extensors, axial and abaxial extensor branches of the interosseous ligament, and the proximal accessory paradigital ligament of the dewclaw insert on the dorsal surface of the body of the second phalanx at the extensor junction. This is a diffuse insertion site, much less discreet than that of the flexor tuberosity on the plantar side, where the superficial flexor tendon inserts, and the insertion site forms a noticeable trapezoidal platform. The medial and lateral digital extensor tendons bifurcate, with one branch inserting along the distal dorsal aspect of the second phalanx, and the other branch continuing to the third phalanx. The abaxial and axial extensor branches likewise partially insert at the extensor junction, then divide again, and continue to the third phalanx. Just below the pastern joint is a large interdigital ligament which is cruciate in form, which extends across the entire axial surface of the second phalanx and inserts at and just distal to the coffin joint on the second and third phalanges. This large, strong structure is without elasticity, providing protection against over-abduction of the hoof.

## Exocrine gland

Between the pastern joint and the distal cruciate interdigital ligament a large exocrine gland is housed, present in both the fore- and hindlimb. This gland is also present in other cervids, notably *Odocoileus virginianus*. This interdigital gland secretes kairomones to parasites and antimicrobial substances in *O. virginianus* (Hewitt 2011: 50; Gassett et al. 1996). Reindeer lubricate growing antlers with the gland of the hindlimb, odor traces are also secreted from the glands (Laaksonen 2016). It may serve additional purposes in *Rangifer tarandus*, although more research is necessary; the reindeer use the same paths and they often stop to smell the ground as they are walking, so scent marking via the hooves is an area that deserves more research. The pelage of *Rangifer tarandus* additionally increases in length adjacent to the capsule of the hoof, providing a ring of longer hairs around the junction between hide and cartilage at the hoof proper as well as the hoof capsules of the dewclaws. These differences and microscopic analysis of pelage are detailed by Zhang et al. (2019).

## Dorsal interphalangeal joint of both fore- and hindlimb (coffin joint)

The distal interphalangeal joint, or coffin joint, is covered by the transition from the dermis to the cartilaginous hoof capsule (Fig. 7). This joint marks the terminus of most of the tendons and ligaments of the hoof. The third phalanx is pyramidal in shape, with dorsal, plantar, and axial sides, as well as coronary, soleal, and dorsal borders. It also contains an axial foramen, just proximal to the extensor process, and an abaxial foramen on the abaxial palmar surface proximal to the soleal border. The dorsal interphalangeal collateral ligaments run from the concave surfaces on the axial and abaxial sides of the head of the second phalanx. The axial dorsal interphalangeal collateral ligament partially divides into two branches, both inserting along the axial border of the dorsal aspect of the third phalanx. The abaxial dorsal interphalangeal collateral ligament inserts along the coronary border to the abaxial side of the dorsal border.

The abaxial digital extensor and the proximal accessory digital extensor of the dewclaw partially combine with the other digital extensors at the pastern joint, but both also have branches running to the third phalanx. Both curl around the second phalanx after partially inserting on the dorsal surface, terminating on the plantar articular border of the third phalanx. Finally, the distal accessory paradigital ligament from the dewclaw extends to insert at the parietal groove on the abaxial surface of the third phalanx.

The long, thin, fibrous common digital extensor tendon of the forelimb and the long digital extensor tendon of the hindlimb insert on the extensor tubercle, an ovoid osseous structure at the proximal dorsal axial part of the third phalanx. Abaxial to this, along the border of the articular surface of the dorsal axial aspect of the third phalanx, the long branches of the medial and lateral digital extensor tendons insert on a small ridge of bone on the proximal edge of the abaxial surface. A small sesamoidal ligament runs from the proximal aspect distal sesamoid to the third phalanx, while the impar ligament runs from the distal aspect of the distal sesamoid to the third phalanx, further securing it in place. The deep digital flexor, after partially inserting at the flexor tubercle and passing over the distal sesamoid, fans out to make a diffuse insertion across nearly the entirety of the plantar surface of the third phalanx.

## Additional aspects of the hoof

Compared to the rest of the metapodial and digits, the hoof capsule is much more densely packed with vascularized soft tissue structures. The surface of the hoof is divided into several parts. As per the definitions given in (Budras et al. 2003; Habel 1949; McLeod 1956) for domestic cattle, the perioplic segment which defines the edge between the hide and the hoof is a slightly thicker band in *Rangifer tarandus*, which then gives way to the coronary segment, extending down the rest of the dorsal portion of the hoof. The sole segment covers the cranial point of the hoof, while the bulbar segment covers most of the plantar surface of the hoof and covers the hoof bulb, internally. The hoof bulb is a highly vascularized cushion of soft tissue covering the caudal portion of the internal hoof, running superficially to the deep digital flexor. This hoof bulb is innervated and vascularized by nerves, veins, and arteries running the length of the metapodials and digits to branch out into a network in the hoof capsule, with veins and arteries running through the abaxial and axial foramina of the third phalanx.

## Discussion

The description of these structures in the hoof of *Rangifer tarandus* has implications for multiple fields, including wildlife biology, zooarchaeology, and veterinary science, but also providing resources for those who work closely with reindeer and caribou. In wildlife biology, any greater understanding of how the animal body functions can help inform studies of behavior, migration, foraging, and herd interactions by helping indicate where stress and injury may occur internally. Likewise, in veterinary science, a knowledge of the internal structures of reindeer feet may help veterinarians and herders give more precise and accurate medical care to ailing domestic animals, leading to a better quality of life.

For zooarchaeologists, understanding the points of attachment and stress, as well as the associated physiology, opens up many lines of inquiry. An understanding of tendinous and ligamentary insertions may help identify points of entheseal change, which may in turn help identify patterns of habitual activity (Hull et al. 2020; Salmi et al. 2020, Villotte and Knüsel 2013; Weiss et al. 2012). For skeletal specimens, entheseal changes can be informative in the identification of ecotype, foraging pattern, and even identification of domestic animals, particularly those that are working animals (Niinimäki and Salmi 2013; Salmi and Niinimäki 2016). Lastly, the understanding of these soft tissue structures may help scholars contextualize butchery marks and how they relate to conversion of reindeer bodies into specific products.

*Rangifer tarandus* hooves are similar to the feet of other ungulates in several ways. The large, basic forms of flexor, extensor, and collateral tendons and ligaments, while slightly different in placement, are analogous to those other artiodactyls, especially *Bos taurus,* the domestic cow, for which a great deal of veterinary literature exists. The greatest differences in foot anatomy between *Bos taurus* and *Rangifer tarandus* are seen in the form and function of the dewclaws. The vestigial MC/MT V (but not II) in *Bos taurus* is represented by the stifle, which descends from the proximal end of the metapodial, while the bones of the dewclaw are represented by one or two tiny bones (Budras and Habel 2011; Habel 1949; McLeod 1956). These dewclaws are non-functioning and rarely make contact with the ground. By contrast, the dewclaw of *Rangifer tarandus* is highly functional, especially in the forelimb, where the vestigial MC II/V form stylets which are connected by branches of tendons and activate in concert with the digits proper. The bones of the dewclaw in both fore- and hindlimb have identifiable first, second, and third phalanges. In addition, the strong accessory abaxial extensor tendon branches of the fore- and hindlimb dewclaw act to stabilize the hoof and prevent over-abduction. This creates a much larger hoof surface for locomotion and foraging. This unique adaptation shows the efficiency of the *Rangifer tarandus* body in snowy environments, but this may also open the hoof up to different injury and stress patterns compared to feet of other ungulates, a subject which has been explored in other species in zooarchaeology (Izeta 2008, Izeta and Cortés 2006).

This study is limited, as it was done only on the limbs of a small number of domestic reindeer, and therefore may not represent the full range of variation present in the global populations of this species. Further research into the hoof anatomy that includes other subspecies of *Rangifer tarandus* such as *R.t. caribou* and *R.t. granti* in North America and high Arctic ecotypes of the far North such as *R.t. platyrhynchus* would further expand our understanding of *Rangifer tarandus* anatomy. While this study provides only the basic anatomy of *Rangifer tarandus* hooves, it is hoped that this research will lead to more understanding of reindeer and caribou anatomy and assist in answering questions in both the past and present.
